# B Cell Reconstitution is Associated With COVID-19 Booster Vaccine Responsiveness in Patients Previously Seronegative Treated With Rituximab

**DOI:** 10.3899/jrheum.220475

**Published:** 2022-12-15

**Authors:** Kaitlin Schultz, Deanna Jannat-Khah, Robert Spiera

**Affiliations:** 1 Department of Medicine, Hospital for Special Surgery, Weill Cornell Medical College, New York, New York, USA.; 2 Department of Medicine, Hospital for Special Surgery, and Department of Medicine, Weill Cornell Medical College, New York, New York, USA.

**Keywords:** B lymphocytes, rheumatic diseases, vaccination

## Abstract

**Objective.:**

To assess factors associated with serologic response to the coronavirus 2019 (COVID-19) booster vaccine in patients with autoimmune rheumatic diseases treated with rituximab (RTX) who were previously serologically unresponsive to the initial vaccine series.

**Methods.:**

A retrospective chart review of patients treated with RTX who failed to demonstrate a serologic response to the first SARS-CoV-2 vaccination series and subsequently received an mRNA vaccine booster was performed. Serologic response ≥ 4 weeks after the booster was the primary outcome. Fisher exact tests, *t* tests, and Wilcoxon rank-sum tests were used for comparisons.

**Results.:**

In 31 patients who were previously seronegative, 68% seroconverted following a booster of the COVID-19 vaccine. B cell reconstitution was significantly different between those with positive (median 1.79, IQR 0.65–3.00) and negative (median 0, IQR 0–0) serologic responses to the booster. The days from last RTX dose were also statistically different among seroconverters (median 301, IQR 251–368) vs nonseroconverters (median 188, IQR 169–245). Demographic characteristics were not associated with serologic positivity. Positive predictive value of B cell presence was 90.9% (95% CI 70.8–98.9) and negative predictive value was 100% (95% CI 59–100) for serologic response to the mRNA booster vaccine. Positive predictive value of time ≥ 6 months from last RTX dose to booster was 78.3% (95% CI 56.3–92.5) and the negative predictive value was 62.5% (95% CI 24.5–91.5).

**Conclusion.:**

Detectable B cells and longer time from last RTX exposure were associated with the development of anti-SARS-CoV-2 spike protein antibodies following the booster vaccine. These findings should be considered in timing boosters in patients treated with RTX.

Vaccination has emerged as a cornerstone of protection against severe outcomes of SARS-CoV-2 infection, which is relevant to patients with autoimmune rheumatic diseases (AIRDs). Compromised serologic response to COVID-19 vaccines has been demonstrated in patients treated with immunosuppressive therapies, including rituximab (RTX).^1^ Current guidelines for vaccination in patients treated with B cell depleting agents offer inconsistent recommendations. The American College of Rheumatology (ACR) guidelines suggest inoculating patients 4 weeks prior to subsequent dosing of RTX.^2^ Although time from last RTX dose is not specified, the interval between RTX doses is commonly 6 months for remission maintenance in rheumatoid arthritis (RA) and antineutrophil cytoplasmic antibody–associated vasculitis (AAV). However, the European Alliance of Associations for Rheumatology and the Canadian Rheumatology Association do not discuss timing nor B cell reconstitution.^3,4^ Several studies have suggested that B cell reconstitution after RTX treatment is associated with a greater likelihood of positive serologic response to the initial vaccine series.^5–8^ ACR guidelines mention consideration of B cell reconstitution for vaccine administration in patients treated with RTX, although no formal recommendation was suggested.^2^

Administration of additional doses of the COVID-19 vaccine has emerged as a strategy for continued protection against SARS-CoV-2.^9^ This strategy has proven to be safe in patients treated with RTX, but often has not resulted in seroconversion in patients who were seronegative following the initial vaccine series.^10^ Moreover, there is a paucity of data regarding factors associated with successful seroconversion following a booster dose in patients treated with RTX. In the observational studies specifically on patients with AIRD treated with RTX reported to date, anywhere from 0% to 47% of patients that were previously seronegative were able to develop antibodies after the booster.^10–18^ The study that achieved a seroconversion rate of 47% only included 15 patients.^11^ In 3 of these studies, a positive association was found between exhibiting a positive CD19% and vaccine response.^11–13^ The other studies either did not assess B cell reconstitution or were limited by small numbers (< 10) of patients who seroconverted.^10,14–18^ To better understand the relationship between the third dose of the mRNA COVID-19 vaccine with seroconversion among patients treated with RTX, we retrospectively assessed factors associated with COVID-19 vaccine response in 31 patients with AIRD treated with RTX who had not demonstrated a serologic response to their initial COVID-19 vaccine series.

## METHODS

A retrospective chart review of 31 adult patients who were inoculated with a booster of an mRNA COVID-19 vaccine (Pfizer of Moderna) was performed. All 31 patients had a clinic visit between February 24, 2021, and November 17, 2021, at 1 rheumatology practice. Patients did not provide written consent as this was a retrospective review. Patients treated with RTX were included if they were seronegative following the first series of the COVID-19 vaccine as measured by either the Elecsys Anti-SARS-CoV-2 (Roche) or Healthineers SARS-CoV-2 Total Assay Atellica IM (Siemens) and subsequently received a booster dose. Following standard practice in the United States, we defined the first 2 doses of the COVID-19 vaccine as the initial series and the third dose of the vaccine as the booster. Our cohort included 3 patients treated with RTX between their second and third dose of the COVID-19 vaccine. All other patients had their last infusion prior to their first vaccine dose. There was no specific RTX scheme used in the study as all patients were being treated according to standard care. Patients that experienced a known prior COVID-19 infection were excluded. The Hospital for Special Surgery Institutional Review Board approved the study (IRB #2021–0480).

The primary outcome was serologic response to the COVID-19 spike protein evaluated using either Roche (n = 26; specificity 99.8%, sensitivity 99.5%) or Siemens (n = 5; specificity 99.82%, sensitivity 100%) measured at ≥ 4 weeks after the booster was administered. The patient was deemed seropositive according to the normal ranges for each specific assay. The threshold used was > 0.4 U/mL on the Roche assay and > 1 U/mL on the Siemens assay. CD19 positive cell percentage taken near the date of the booster was used to define B cell reconstitution. Other data collected included patients’ demographics, diagnoses, date, and type of all vaccines, date of their last RTX dose, Ig levels, absolute lymphocyte levels, and concomitant antirheumatic therapy.

Descriptive statistics were calculated, and comparisons were assessed by *t* test, Fisher exact test, and Wilcoxon rank-sum test. Box and whisker plots were created to display differences between the time from the last RTX infusion to booster dose and the percentage of B cell reconstitution between patients who were seropositive and seronegative. Positive and negative predictive values were calculated to assess serologic response and the status of B cell reconstitution and serologic response and the time from the last RTX dose to booster dose. Stata version 14.0 (StatCorp) was used and an α of 0.05 defined statistical significance.

## RESULTS

Thirty-one patients treated with RTX were included. Seventy-four percent of patients (n = 23) were female, with a median age of 61.5 (SD 16.7; Table). The majority of patients were White (87%, n = 27) and were being treated for granulomatosis with polyangiitis (45%, n = 14). No demographic characteristics were found to be significantly different between patients who were seropositive and seronegative (Table). Corticosteroids (16%, n = 5) were the most prevalent concomitant medication and neither corticosteroid nor other immunosuppressant use differed in those who seroconverted vs those who did not (*P* > 0.99). There was a slight difference between seroconverters (median 750, IQR 500–1000) and nonseroconverters (median 1000, IQR 1000–1000) for the most recent RTX dose; however, it was not statistically significant (*P* = 0.08). The median for the number of days from the last dose of primary immunization to the third vaccine was 155 (IQR 147–194) for patients who were seronegative and 157 (IQR 138–186) for patients who were seropositive. This difference was also not statistically significant (*P* = 0.66; data not shown).

Absolute lymphocyte levels were not statistically different between vaccine responders and nonresponders (*P* = 0.21; Table). IgG levels were lower in patients without a serologic response than in those who did seroconvert (689 mg/dL, IQR 651–757 vs 928 mg/dL, IQR 735–1001; *P* = 0.02). Twenty-three patients had their B cells measured prior to their booster. Of the remaining 8 patients, 6 patients had their B cells measured at the time of antibody measurement. Three of these patients had a CD19% of 0 and it was assumed that they were B cell–depleted when the booster was administered as they had not received treatment with any B cell–depleting agent between the time of their booster dose and that measurement. A sensitivity analysis was done excluding the remaining 3 patients and no statistically significant differences were found (Supplementary Table S1, available from the authors upon request).

**Figure 1.**
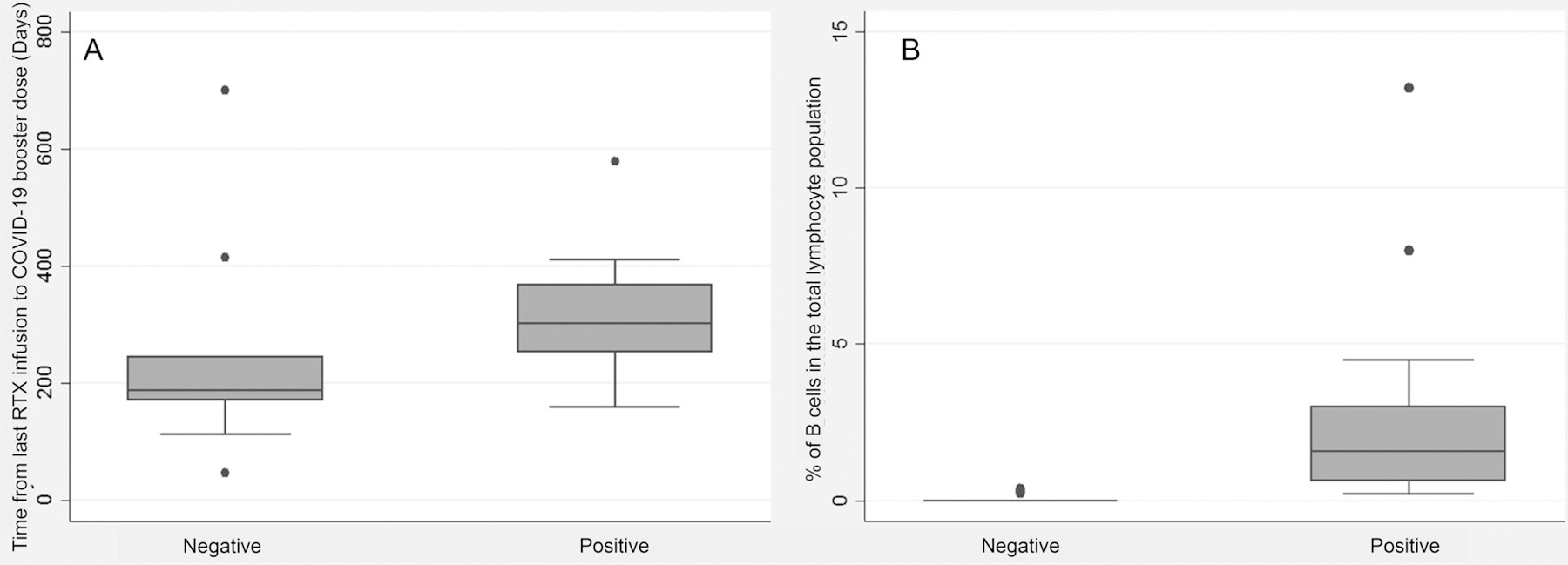
COVID-19 booster response by time from last RTX infusion and B cell status to antibody measurement. (A) Among patients with a negative serologic response, the median days from last infusion to COVID-19 booster vaccination was 188 (IQR 169–245) days. Patients with a positive serologic response had a median of 301 (IQR 251–368) days. Wilcoxon rank-sum test was used to calculate the *P* value. (B) The percentage of B cells among the negative serologic response median was 0 (IQR 0–0). Among the positive serologic vaccine response group, the median was 1.79 (IQR 0.65–3.00). The *P* value is from the Wilcoxon rank-sum test. The y-axis is the percentage of B cells in the total lymphocyte population. COVID-19: coronavirus disease 2019; RTX: rituximab.

Presence of detectable B cells was associated with a positive vaccine response (Figure 1). Ninety-one percent (20/22) of patients with detectable B cells demonstrated a positive serologic response, whereas 0% (0/7) without detectable B cells demonstrated a positive vaccine response (*P* < 0.001; Table). The positive predictive value of B cell reconstitution for COVID-19 serologic response was 90.9% (95% CI 70.8–98.9) and the negative predictive value was 100% (95% CI 59–100).

Time from last RTX dose was different between seroconverters (median days 301, IQR 251–368) vs nonseroconverters (median days 188, IQR 169–245; *P* = 0.03; Table). In patients with < 6 months since last RTX exposure, 63% (5/8) overall remained seronegative following the booster, but among those with B cells beginning to reconstitute in that group, only 25% (1/4) did not seroconvert. In those receiving booster doses 6 to 12 months from last RTX dose, 20% (3/15) did not seroconvert, and in patients receiving boosters > 12 months from last RTX dose, 25% (2/8) remained seronegative. Overall, among those patients receiving booster vaccines > 6 months after last RTX dose and who were beginning to reconstitute B cells, only 6% (1) of patients failed to seroconvert. Positive predictive value of ≥ 6 months from the last RTX dose to the booster was 78.3% (95% CI 56.3–92.5) and the negative predictive value was 62.5% (95% CI 24.5–91.5).

## DISCUSSION

Booster doses of the SARS-CoV-2 vaccines have emerged as an important strategy for containing the pandemic. In our cohort of patients with AIRD treated with RTX who were serologically unresponsive to the initial vaccine series, we found that 32% of patients remained seronegative after the administration of the booster vaccine. CD19% and time from last RTX dose were associated with vaccine responsiveness, whereas demographic characteristics, corticosteroid usage, immunosuppressant usage, time between second and third COVID-19 vaccines, and RTX dosage were not associated with seroconversion.

Despite the lack of SARS-CoV-2 booster studies in patients with AIRD, to date, there are a significant number of studies assessing additional COVID-19 vaccine doses in patients with hematologic malignancy and multiple sclerosis that were receiving anti-CD20 therapy.^19–23^ In a study of 44 patients with B cell non-Hodgkin lymphoma undergoing anti-CD20 therapy that were previously unresponsive to the first vaccine series, 13 (30%) seroconverted following the booster. Although the authors did not directly assess B cells, they identified that greater time from last anti-CD20 therapy and higher absolute lymphocyte counts were associated with a greater likelihood of seroconversion.^19^ In contrast, a study of 16 patients with multiple sclerosis being treated with either ocrelizumab or RTX reported that only 1 patient developed significant measurable antibodies to the SARS-CoV-2 booster. This patient was also the only one that had detectable B cells prior to the third dose of the vaccine.^20^ A third study looked at COVID-19 vaccine booster efficacy in patients with immune-mediated inflammatory disorders. Thirty-one of 68 (45.6%) patients that were being treated with an anti-CD20 therapy were able to seroconvert after their third vaccination. Although this population included patients with AIRD, the authors do not specify how many had rheumatic diseases and did not discuss timing from last anti-CD20 therapy or B cell counts.^23^

Strategies to optimize serologic response to boosters of the vaccines specifically in patients with AIRD treated with RTX are of importance as B cell depletion has been associated with worse outcomes from COVID-19.^24^ A study assessing COVID-19 infections in patients post vaccination found that patients treated with RTX had higher rates of hospitalization than patients being treated with other immunosuppressants.^25^ Longer time from last RTX administration and the presence of B cells have emerged as the features most predictive of a serologic response to the initial vaccine series.^5–8^

There is a paucity of data regarding factors associated with a serologic response to COVID-19 vaccines boosters in patients treated with RTX who were initially seronegative. In the largest study to date, only 14.5% (9/62) of patients seroconverted following a third dose. The authors did not measure B cell status and, therefore, do not have data on the relevance of B cell reconstitution to likelihood of a serologic response.^16^ Similarly, another study found that only 27% (15/55) of patients treated with RTX developed antibodies following the booster. The authors identified that B cells were predictive of vaccine response as 67% (12/18) of patients with detectable B cells became seropositive following the booster. However, the authors did not discuss time from last RTX dose.^12^ In a study that included 49 patients treated with RTX, only 16.3% (8/49) seroconverted following the booster, and no association was found with either B cell levels or time from last RTX dose.^14^ The relatively small numbers of patients who seroconverted in that study likely contributed to the inability to recognize positive associations. Similarly, a study that included 33 patients treated with RTX demonstrated only an 18.2% (6/33) rate of seropositivity following the booster; however, CD19% association was not analyzed.^10^ A smaller study that did assess B cell reconstitution found that 7 patients who were previously seronegative became serologically positive following the booster, and 5 of those patients had reconstituted B cells.^11^ Two additional studies included patients with AIRD treated with RTX as part of their larger cohorts, but once again had a small proportion of patients become seropositive and did not report on B cell levels.^17–18^ Last, in 2 smaller studies, among 13 patients, only 1 patient who was B cell depleted at the administration of the third dose demonstrated seroconversion.^13,15^

In our study, a higher percentage (68%, 21/31) of patients developed antibodies after the booster who were previously seronegative after the initial vaccine series. We found that detectable B cells (*P* < 0.001) and time from last RTX exposure (*P* = 0.03) were strongly associated with a positive response. This observation could help inform strategies for optimizing response to booster COVID-19 vaccine in patients treated with RTX. In our patients > 6 months from their last RTX dose, the interval suggested in some guidelines, more than a fifth (22%, 5/23) remained seronegative following the booster. Complementary information regarding B cell reconstitution could better inform if the patient will respond to the vaccine, as only 6% of patients beginning to reconstitute B cells in that group were seronegative following the booster.

A strength of our study includes the much higher proportion of patients who successfully seroconverted following booster vaccine administration. This allowed us to recognize associations with factors indicative of a greater likelihood of seroconversion after administration of a booster dose of the COVID-19 vaccine than what could be recognized in smaller studies, or those with low rates of seroconversion. Another strength is that we specifically focused on patients with AIRD treated with RTX who had not seroconverted following their initial vaccine series. The majority of the existing literature has this patient population included as part of a larger heterogeneous cohort that includes other diagnoses. Among the patients with AIRD that are included in these studies, most are being treated with RTX for RA. Contrastingly, the majority of our patients were being treated for AAV (16/31, 52%). The standard treatment for RA often includes the concomitant use of methotrexate, whereas AAV does not. Therefore, our results may be less affected by this confounding variable than the current studies.

A limitation of our study is its retrospective nature and lack of non-RTX–treated controls. Although all COVID-19 spike antibodies were measured more than 4 weeks after booster vaccine administration, the timing of that measurement was not standardized. Antinucleocapsid antibodies were also not captured to assess prior COVID-19 infection as this was not part of standard care; however, patients with a known history of COVID-19 infection were excluded. Moreover, data on B cell status were measured after booster vaccination in 6 patients. Three patients remained B cell depleted at the time of antibody measurement and had not received additional RTX, so they were assumed to be B cell–depleted at the time of vaccination. A sensitivity analysis excluding the remaining 3 patients who had detectable B cells after the booster did not change the results. Although concomitant medications and corticosteroid use were not associated with differential vaccine responses, this could be a result of our small sample size. Differences reported between those 6 to 12 months and those greater than 12 months from their last RTX dose could also be limited by the small sample size of each respective cohort. Further, persistent B cell depletion may relate to more severe or tenuous underlying disease and more prior immunosuppression, although differences in disease severity were not apparent. Finally, the clinical implications of serologic positivity for COVID-19 spike antibodies are uncertain. Evidence of T cell mediated immunity to SARS-CoV-2 in vaccinated patients previously treated with RTX has been demonstrated.^9–10,14^ Larger prospective studies assessing responses to booster COVID-19 vaccines are needed to identify factors associated with seropositivity and more importantly, protection from severe clinical outcomes. This study, however, suggests that incorporating assessment of B cell reconstitution into clinical decisions on timing of booster doses should be considered.

## Figures and Tables

**Table.** Bivariate comparisons between patients who were seronegative and seropositive to the COVID-19 booster by characteristics and demographics.

	Value	Negative	Positive	*P* *
N	31	10	21	
Age, mean (SD)	61.5 (16.7)	62.3 (14.0)	61.0 (18.1)	0.85
Age, median (IQR)	64 (51–72)	63 (51–69)	65 (51–73)	0.75
Sex, n (%)				> 0.99
Female	23 (74)	7 (70)	16 (76)	
Male	8 (26)	3 (30)	5 (24)	
Race, n (%)				0.49
White	27 (87)	8 (80)	19 (90)	
Black or African American	1 (3)	1 (10)	0 (0)	
Asian	3 (10)	1 (10)	2 (10)	
RA, n (%)	4 (13)	1 (10)	3 (14)	> 0.99
Sjögren syndrome, n (%)	5 (16)	2 (20)	3 (14)	> 0.99
SSc, n (%)	3 (10)	1 (10)	2 (10)	> 0.99
GPA, n (%)	14 (45)	5 (50)	9 (43)	> 0.99
PMR, n (%)	1 (3)	0 (0)	1 (5)	> 0.99
IgG4 disease, n (%)	1 (3)	1 (10)	0 (0)	0.32
MPA, n (%)	2 (6)	0 (0)	2 (10)	> 0.99
IgG levels around booster dose, mg/dL,median (IQR)	757 (656–963)	689 (651–757)	928 (735–1001)	0.02
IgG levels around booster dose, n (%)				0.32
< 400 mg/dL	1 (3)	1 (10)	0 (0)	
< 700 mg/dL	9 (29)	5 (50)	4 (19)	
Absolute lymphocyte levels around booster dose, median (IQR)	1.6 (1.3–1.9)	1.4 (1.1–1.7)	1.7 (1.4–1.9)	0.21
Antirheumatic therapies other than RTX				
Any immunosuppressant, n (%)	9 (29)	3 (30)	6 (29)	> 0.99
LEF	1 (3)	0 (0)	1 (5)	> 0.99
AZA	1 (3)	1 (10)	0 (0)	0.32
MTX	3 (10)	2 (20)	1 (5)	0.24
MMF	3 (10)	0 (0)	3 (14)	0.53
TCZ	1 (3)	0 (0)	1 (5)	> 0.99
CS, n (%)	5 (16)	1 (10)	4 (19)	> 0.99
Prednisone dose, mg, median (IQR)	4.8 (4.0–5.5)	5.0 (5.0–5.0)	4.5 (3.5–6.0)	0.65
First vaccine series type, n (%)				0.29
Pfizer	18 (58)	8 (80)	10 (48)	
Moderna	11 (35)	2 (20)	9 (43)	
J&J	1 (3)	0 (0)	1 (5)	
Sinovac	1 (3)	0 (0)	1 (5)	
Third vaccine dose type, n (%)				0.24
Pfizer	19 (61)	8 (80)	11 (52)	
Moderna	12 (39)	2 (20)	10 (48)	
Dichotomous B cell status around booster dose, n (%)				< 0.001
No detectable B cells	7 (23)	7 (70)	0 (0)	
Detectable B cells	22 (71)	2 (20)	20 (95)	
Missing	2 (6)	1 (10)	1 (5)	
RTX for remission induction, n (%)	2 (6)	0 (0)	2 (10)	> 0.99
Time from last RTX infusion to booster, days, median (IQR)	260 (216–379)	188 (169–245)	301 (251–368)	0.03
Time from last RTX infusion to booster dose, months, n (%)				0.10
< 6	8 (26)	5 (50)	3 (14)	
6–12	15 (48)	3 (30)	12 (57)	
> 12	8 (26)	2 (20)	6 (29)	

* *P* values are from Fisher exact tests, *t* tests, and Wilcoxon rank sum tests. AZA: azathioprine; CS: corticosteroid; COVID-19: coronavirus disease 2019; GPA: granulomatosis with polyangiitis; LEF: leflunomide; MMF: mycophenolate mofetil; MPA: microscopic polyangiitis; MTX: methotrexate; PMR: polymyalgia rheumatica; RA: rheumatoid arthritis; RTX: rituximab; SSc: systemic sclerosis; TCZ: tocilizumab.

## References

[1] Spiera R, Jinich S, Jannat-Khah D. Rituximab, but not other antirheumatic therapies, is associated with impaired serological response to SARS-CoV-2 vaccination in patients with rheumatic diseases. Ann Rheum Dis 2021;80:1357–9. 33975857 10.1136/annrheumdis-2021-220604

[2] Curtis JR, Johnson SR, Anthony DD, American College of Rheumatology guidance for COVID-19 vaccination in patients with rheumatic and musculoskeletal diseases: version 3. Arthritis Rheumatol 2021;73:e60–75. 34346564 10.1002/art.41928 PMC8426685

[3] EULAR. EULAR view-points on SARS-CoV-2 vaccination in patients with RMDs [Internet. Accessed December 6, 2022.] Available from: https://www.eular.org/eular_sars_cov_2_vaccination_rmd_patients.cfm

[4] Hazlewood GS, Pardo JP, Barnabe C, Canadian Rheumatology Association recommendation for the use of COVID-19 vaccination for patients with autoimmune rheumatic diseases. J Rheumatol 2021;48:1330–9. 33993119 10.3899/jrheum.210288

[5] Stefanski A, Rincon-Arevalo H, Schrezenmeier E, B cell numbers predict humoral and cellular response upon SARS-CoV-2 vaccination among patients treated with rituximab. Arthritis Rheumatol 2022;74:934–47. 34962360 10.1002/art.42060 PMC9011692

[6] Jinich S, Schultz K, Jannat-Khah D, Spiera R. B cell reconstitution is strongly associated with COVID-19 vaccine responsiveness in rheumatic disease patients who received treatment with rituximab. Athritis Rheumatol 2022;74:776–82. 10.1002/art.42034 34908241

[7] Mrak D, Tobudic S, Koblischke M, SARS-CoV-2 vaccination in rituximab-treated patients: B cells promote humoral immune responses in the presence of T-cell-mediated immunity. Ann Rheum Dis 2021;80:1345–50. 34285048 10.1136/annrheumdis-2021-220781

[8] Moor M, Suter-Riniker F, Horn M, Humoral and cellular responses to mRNA vaccines against SARS-CoV-2 in patients with a history of CD20 B-cell-depleting therapy (RituxiVac): an investigator-initiated, single-centre, open-label study. Lancet Rheumatol 2021;3:e789–97. 34514436 10.1016/S2665-9913(21)00251-4 PMC8423431

[9] Benotmane I, Gautier G, Perrin P, Antibody response after a third dose of the mRNA-1273 SARS-CoV-2 vaccine in kidney transplant recipients with minimal serologic response to 2 doses. JAMA 2021;326:1063–5. 34297036 10.1001/jama.2021.12339 PMC8456389

[10] Simon D, Tascilar K, Fagni F, Efficacy and safety of SARS-CoV-2 revaccination in non-responders with immune-mediated inflammatory disease. Ann Rheum Dis 2022; 81:1023–1027. 34819271 10.1136/annrheumdis-2021-221554

[11] Kant S, Azar A, Geetha D. Antibody response to COVID-19 booster vaccine in rituximab-treated patients with anti-neutrophil cytoplasmic antibody-associated vasculitis. Kidney Int 2022;101:414–5. 34822874 10.1016/j.kint.2021.11.012 PMC8607746

[12] Bonelli M, Mrak D, Tobudic S, Additional heterologous versus homologous booster vaccination in immunosuppressed patients without SARS-CoV-2 antibody seroconversion after primary mRNA vaccination: a randomised controlled trial. Ann Rheum Dis 2022;81:687–94. 35027397 10.1136/annrheumdis-2021-221558

[13] Felten R, Gallais F, Schleiss C, Cellular and humoral immunity after the third dose of SARS-CoV-2 vaccine in patients treated with rituximab. Lancet Rheumatol 2022;4:e13–6. 34778844 10.1016/S2665-9913(21)00351-9 PMC8575482

[14] Jyssum I, Kared H, Tran TT, Humoral and cellular immune responses to two and three doses of SARS-CoV-2 vaccines in rituximab-treated patients with rheumatoid arthritis: a prospective, cohort study. Lancet Rheumatol 2022;4:e177–87. 34977602 10.1016/S2665-9913(21)00394-5 PMC8700278

[15] Kant S, Geetha D. Impact of rituximab on humoral response to COVID-19 booster vaccine and antibody kinetics in patients with anti-neutrophil cytoplasmic antibody vasculitis. Kidney Int 2021;100:1124–7. 34499910 10.1016/j.kint.2021.08.020 PMC8421079

[16] Bitoun S, Avouac J, Henry J, Very low rate of humoral response after a third COVID-19 vaccine dose in patients with autoimmune diseases treated with rituximab and non-responders to two doses. RMD Open 2022;8:e002308. 35589332 10.1136/rmdopen-2022-002308 PMC9121105

[17] Aikawa NE, Kupa LVK, Silva CA, Strong response after fourth dose of mRNA COVID-19 vaccine in autoimmune rheumatic diseases patients with poor response to inactivated vaccine. Rheumatology 2023;62:480–5. 10.1093/rheumatology/keac301 PMC9213862 35639644

[18] Sidler D, Born A, Schietzel S, Trajectories of humoral and cellular immunity and responses to a third dose of mRNA vaccines against SARS-CoV-2 in patients with a history of anti-CD20 therapy. RMD Open 2022;8:e002166. 35361691 10.1136/rmdopen-2021-002166 PMC8971359

[19] Avivi I, Luttwak E, Saiag E, BNT162b2 mRNA COVID-19 vaccine booster induces seroconversion in patients with B-cell non-Hodgkin lymphoma who failed to respond to two prior vaccine doses. Br J Haematol 2022;196:1329–33. 35075635 10.1111/bjh.18029

[20] Achtnichts L, Jakopp B, Oberle M, Humoral immune response after the third SARS-CoV-2 mRNA vaccination in CD20 depleted people with multiple sclerosis. Vaccines 2021;9:1470. 34960216 10.3390/vaccines9121470 PMC8707582

[21] Greenberger LM, Saltzman LA, Senefeld JW, Johnson PW, DeGennaro LJ, Nichols GL. Anti-spike antibody response to SARS-CoV-2 booster vaccination in patients with B cell-derived hematologic malignancies. Cancer Cell 2021;39:1297–9. 34509182 10.1016/j.ccell.2021.09.001 PMC8421105

[22] Azzolini E, Pozzi C, Germagnoli L, mRNA COVID-19 vaccine booster fosters B- and T-cell responses in immunocompromised patients. Life Sci Alliance 2022;5:e202201381. 35169017 10.26508/lsa.202201381 PMC8860093

[23] Wieske L, van Dam KPJ, Steenhuis M, Humoral responses after second and third SARS-CoV-2 vaccination in patients with immune-mediated inflammatory disorders on immunosuppressants: a cohort study. Lancet Rheumatol 2022;4:e338–50. 35317410 10.1016/S2665-9913(22)00034-0 PMC8930018

[24] Levavi H, Lancman G, Gabriolove J. Impact of rituximab on COVID-19 outcomes. Ann Hematol 2021;100:2805–12. 34549309 10.1007/s00277-021-04662-1 PMC8455155

[25] Boekel L, Stalman E, Wieske L, Breakthrough SARS-CoV-2 infections with the delta (B.1.617.2) variant in vaccinated patients with immune-mediated inflammatory diseases using immunosuppressants: a substudy of two prospective cohort studies. Lancet Rheumatol 2022;4:e417–29. 35527808 10.1016/S2665-9913(22)00102-3 PMC9054068

